# *In vitro* interaction of lumefantrine and piperaquine by atorvastatin against *Plasmodium falciparum*

**DOI:** 10.1186/1475-2875-13-189

**Published:** 2014-05-25

**Authors:** Jérome Dormoi, Hélène Savini, Rémy Amalvict, Eric Baret, Bruno Pradines

**Affiliations:** 1 Unité de Parasitologie et d’Entomologie, Département de Microbiologie, Institut de Recherche Biomédicale des Armées, Brétigny sur Orge, France; 2 Unité de Parasitologie, Département d’Infectiologie de Terrain, Institut de Recherche Biomédicale des Armées, Marseille, France; 3 Unité de Recherche sur les Maladies Infectieuses et Tropicales Emergentes, UM 63, CNRS 7278, IRD 198, Inserm 1095, Aix Marseille Université, Marseille, France; 4 Service des Maladies Infectieuses, Hôpital d’Instruction des Armées Laveran, Marseille, France; 5 Centre National de Référence du Paludisme, Marseille, France

**Keywords:** Malaria, *Plasmodium falciparum*, Anti-malarial, Resistance, *In vitro*, statin

## Abstract

**Background:**

There is an urgent need for the discovery of new anti-malarial drugs and combination therapy. A combinatorial approach protects each drug from the development of resistance and reduces generally the overall transmission rate of malaria. Statins, the inhibitors of 3-hydroxy-3-methylglutaryl-Coenzyme A reductase and a family of lipid-lowering drugs, have *in vitro* anti-malarial properties, and more specially atorvastatin. However, atorvastatin has a short elimination half-life (14 hours) and an efficient combination of anti-malarial drugs must associate a drug with a short elimination half-life and a drug with a long elimination half-life. The objective of the present work was to identify new potential partners among standard new anti-malarial drugs with long elimination half-life, such as lumefantrine, piperaquine, pyronaridine and atovaquone, to improve the *in vitro* activity of atorvastatin against different *Plasmodium falciparum* strains to treat uncomplicated malaria.

**Methods:**

*In vitro* interaction of atorvastatin in combination with lumefantrine, piperaquine, pyronaridine and atovaquone was assessed against 13 *P. falciparum* strains by isotopic test.

**Results:**

Atorvastatin showed additive effects with pyronaridine, piperaquine and lumefantrine. Atorvastatin increased the *in vitro* activity of lumefantrine and piperaquine at concentrations expected in clinical observations. The average IC_50_ values of lumefantrine decreased significantly from 31.9 nM to 20.5 nM (a decrease of 35.7%) in combination with 1 μM of atorvastatin.

**Conclusions:**

Even though *in vitro* data indicate that atorvastatin improved the activity of lumefantrine and piperaquine, the same may not necessarily be true *in vivo*. Piperaquine, a new drug with long terminal elimination half-life, is currently a very promising anti-malarial drug.

## Background

Over the past 20 years, many strains of *Plasmodium falciparum* have become resistant to chloroquine and other anti-malarial drugs [1]. In 2002, the World Health Organization (WHO) recommended that artemisinin-based combination therapy (ACT) be used to treat all cases of uncomplicated malaria and that artesunate should be used as the first-line treatment for severe malaria in 2011. Several recent studies have reported clinical failures or extended parasite clearance times in Cambodia [2-4].

There is an urgent need for the discovery of new anti-malarial drugs and combination therapy. A combinatorial approach protects each drug from the development of resistance and reduces generally the overall transmission rate of malaria [5].

Statins, the inhibitors of 3-hydroxy-3-methylglutaryl-Coenzyme A reductase (HMG-CoA reductase) and a family of lipid-lowering drugs, have *in vitro* anti-malarial properties [6,7]. Moreover, atorvastatin improved the *in vitro* activity of mefloquine [8], quinine [9], dihydroartemisinin [10] and Proveblue®, a methylene blue preparation that complies with the European Pharmacopoeia and contains limited organic impurities and heavy metals of recognized toxicity [11], at the plasma concentrations expected in clinical observations in patients taking 80 mg of atorvastatin daily (0.1 to 0.5 μM) [12]. However, atorvastatin used alone failed to prevent death from cerebral malaria or to affect the parasitaemia of infected mice [13-15]. Atorvastatin combined with mefloquine (a long half-life drug) led to a significant delay in mouse death and had an effect on the onset of cerebral malaria symptoms [16]. However, the mice died of severe anaemic malaria. The combination of dihydroartemisinin and atorvastatin was effective as a therapeutic scheme for improving mouse survival but less effective for cytokine modulation, which is associated with protection against cerebral malaria [17]. In a therapeutic intraperitonalscheme, the combination of atorvastatin and dihydroartemisinin *versus* dihydroartemisinin alone resulted in a significant delay in mouse death and had an effect on the onset of cerebral malaria symptoms and on the level of parasitaemia. The experimental conditions did not prevent death. Atorvastatin failed to improve *in vivo* efficacy of quinine in cerebral malaria [17]. Atorvastatin has a short elimination half-life (14 hours) compared to proguanil (24 hours), atovaquone (31–73 hours), chloroquine (two to three days), lumefantrine (four to six days), pyronaridine (six days), mefloquine (six to 41 days), or piperaquine (22 days). A combination of anti-malarial drugs must associate a drug with a short elimination half-life and a drug with a long elimination half-life. However, this is a debatable concept because the long half-life drug is exposed for a significant period of time to select parasite resistance.

The objective of the present work was to identify new potential partners among standard new anti-malarial drugs with long elimination half-life, such as lumefantrine, piperaquine, pyronaridine, and atovaquone, to improve the *in vitro* activity of atorvastatin against different *P. falciparum* strains to treat uncomplicated malaria.

## Methods

### Strains of *Plasmodium falciparum*

Thirteen parasite strains or clones from a wide panel of countries (Cambodia, Cameroon, Gabon, the Gambia, Indochina, Republic of Comoros, Republic of the Congo, Senegal, Sierra Leone, and Uganda) were maintained in culture in RPMI 1640 (Invitrogen, Paisley, UK) supplemented with 10% human serum (Abcys SA, Paris, France) and buffered with 25 mM HEPES and 25 mM NaHCO_3_. Parasites were grown in type A^+^ human red blood cells under controlled atmospheric conditions (10% O_2_, 5% CO_2,_ and 85% N_2_) at 37°C with a humidity of 95%. All strains were synchronized twice with sorbitol before use [18]. Clonality was verified using PCR genotyping of polymorphic genetic markers, msp1, msp2, and microsatellite loci [19,20]. The potentiation evaluation of each strain was assessed in three independent experiments, as previously described [21].

### Drugs

Stock solutions were prepared by dilution in methanol for piperaquine, pyronaridine and atovaquone; and in ethanol for lumefantrine. Final concentrations ranged from 0.05 to 318 nM for lumefantrine, 0.1 to 10,000 nM for atovaquone, 0.16 to 100 nM for pyronaridine and 0.78 to 994 nM for piperaquine. A total of 25 μL of stock solution of each concentration was distributed in duplicate or triplicate into Falcon 96-well flat bottom plates. Plates were dried overnight. Atorvastatin stock solution was prepared extemporately with 1% DMSO (v/v) in RPMI and diluted in sterile distilled water for final concentrations included between 0.006 and 32 μM. Aliquots of 25 μl of atorvastatin solution were distributed into Falcon 96-well plates pre-dosed with anti-malarial agents.

### *In vitro* assay

The 50% inhibitory concentration (IC_50_), i.e., the drug concentration corresponding to 50% of the uptake of 3H-hypoxanthine by the parasites in drug-free control wells, was determined by non-linear regression analysis of log-dose/response curves. Data were expressed as the geometric mean IC_50_, and 95% confidence intervals (95% CIs) were calculated. Each anti-malarial drug was serial diluted and combined with a static concentration of atorvastatin to obtain IC_50_ for each anti-malarial drug. Ten various atorvastatin concentrations were used to construct the isobolograms. Isobolograms were constructed by plotting a pair of fractional IC_50_s for each combination of anti-malarial treatment and atorvastatin. Fractional IC_50_s were calculated by dividing the atorvastatin fixed concentration by its IC_50_ alone, and these data were plotted on the horizontal axis. The corresponding fractional IC_50_s of each classical anti-malarial treatment were calculated by dividing the combined IC_50_s of each classical anti-malarial treatment with the atorvastatin by the IC_50_ of the anti-malarial treatment alone, and these data were plotted on the vertical axis. A straight diagonal isobologram indicates an additive effect. Curves above or below the diagonal indicate antagonistic or synergistic effects, respectively. Results close to the diagonal are considered to be additive.

### Statistical analysis

The effects of atorvastatin at concentrations ranging from 0.1 to 1 μM, without intrinsic effects, were analysed with respect to the lumefantrine, piperaquine, pyronaridine, and atovaquone activity. These concentrations were relevant to atorvastatin plasma concentrations that are achievable in patients taking 80 mg of atorvastatin daily (0.1 to 0.5 μM). Statistical analysis was performed using R software® (version 2.10.1). The global Friedman test was used to compare the medians of different matched groups, and the Wilcoxon test was used to compare the medians of two matched under-groups.

## Results

**Figure 1:**
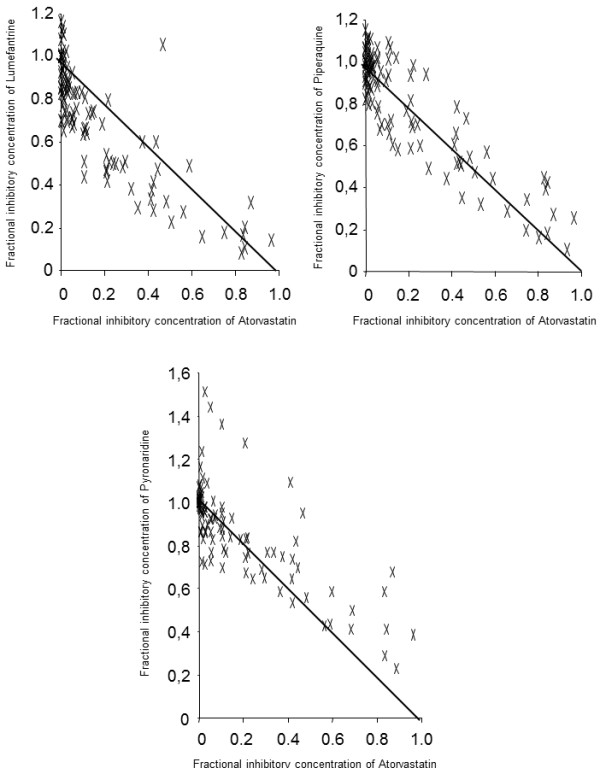
***In vitro *** **combinations of atorvastatin with lumefantrine, piperaquine and pyronaridine against 13 *Plasmodium falciparum *** **strains.**

Atorvastatin showed additive effects with piperaquine, lumefantrine and pyronaridine (Figure 1).

Static concentration of atorvastatin affected significantly the *in vitro* activity of lumefantrine and piperaquine. The average IC_50_ values of lumefantrine decreased significantly from 31.9 nM to 28.1 nM (a decrease of 11%) in combination with 0.12 μM of atorvastatin, 26.3 nM (a decrease of 17.6%) in combination with 0.25 μM of atorvastatin, 24.6 nM (a decrease of 24.8%) in combination with 0.5 μM of atorvastatin and 20.5 nM (a decrease of 35.7%) in combination with 1 μM of atorvastatin, at atorvastatin plasma concentrations expected from clinical observations in patients taking 80 mg of atorvastatin daily (Table 1).

**Table 1:** **Inhibitory activities of lumefantrine, piperaquine, pyronaridine and atovaquone alone and in combination with atorvastatin against 13 strains of *Plasmodium falciparum***

** Anti-malarial drugs**	** Lumefantrine**		** Piperaquine**		** Pyronaridine**		** Atovaquone**	
	**IC**_**50 **_**mean (nM)**	**P-value**	**IC**_**50 **_**mean (nM)**	**P-value**	**IC**_**50 **_**mean (nM)**	**P-value**	**IC**_**50 **_**mean (μM)**	**P-value**
Alone	31.9 ± 15.1		61.1 ± 15.5		30.3 ± 9.6		3.79 ± 2.0	
+ 0.12 μM Atorvastatin	28.1 ± 15.1	0.0068	59.5 ± 19.7	NS	28.9 ± 9.2	NS	3.35 ± 1.5	NS
+ 0.25 μM Atorvastatin	26.3 ± 12.4	0.0019	61.7 ± 18.7	NS	29.1 ± 10.1	NS	3.35 ± 1.6	NS
+ 0.5 μM Atorvastatin	24.6 ± 11.7	0.0019	52.5 ± 17.5	0.0146	28.4 ± 10.2	NS	3.23 ± 1.3	NS
+ 1 μM Atorvastatin	20.5 ± 11.0	0.0009	51.2 ± 19.2	0.0244	26.9 ± 10.0	NS	2.93 ± 1.3	NS

NS if P-value > 0.05.

The average IC_50_ values of piperaquine decreased significantly from 61.1 nM to 52.5 nM (a decrease of 14.1%) in combination with 0.5 μM of atorvastatin and 51.2 nM (a decrease of 16.2%) in combination with 1 μM of atorvastatin.

Atorvastatin did not significantly affect the IC_50_ values of pyronaridine or atovaquone.

## Discussion

Atorvastatin improved strongly the *in vitro* activity of dihydroartemisinin [10], mefloquine [8], quinine [9], and methylene blue [11] and less strongly those lumefantrine and piperaquine. Atorvastatin demonstrated antagonistic effects with anti-malarial drugs whose resistance involves the *P. falciparum* chloroquine resistance transporter gene (*pfcrt*), such as chloroquine, monodesethylamodiaquine [9]. In addition, atorvastatin didn’t affect significantly the *in vitro* activity of pyronaridine whose resistance seems to interact also with *pfcrt* (unpublished observations). A decrease in *P. falciparum* susceptibility to lumefantrine, mefloquine or dihydroartemisinin is associated with polymorphisms in the genes encoding the multidrug resistance (MDR)-like proteins, such as Pgh1, encoded by *pfmdr1* (*P. falciparum* multidrug resistance 1), or in copy numbers of this gene [22-25]. Atorvastatin is an inhibitor of the human phosphoglycoprotein (PgP), an efflux protein involved in cancer cells [26-28]. Pgh1 could be a target for atorvastatin in *P. falciparum* parasites. However, *in vitro* responses to piperaquine seem to be not associated with polymorphisms in *pfcrt* gene [29] and in *pfmdr1*[30] or copy number of *pfmdr1*[31], but associated with repeat polymorphisms in the low-complexity regions of a *P. falciparum* ABC transporter, *pfmdr6*[32]. Atorvastatin seems to improve the *in vitro* activity of anti-malarial drugs whose resistance involves preferentially ABC transporters such as *pfmdr1* and *pfmdr6* and don’t affect or reduce the activity of drugs whose resistance involves preferentially *pfcrt*.

Atorvastatin given with mefloquine and dihydroartemisinin prevents cerebral malaria in a mouse model [16,17]. However, the mice that did not die of cerebral malaria died of severe anaemic malaria. Atorvastatin strongly protects endothelial cells against *P. falciparum*-induced collateral damage, cell apoptosis and endothelial barrier permeabilization [33]. Atorvastatin can be used to reduce *P. falciparum*cyto-adherence to endothelial cells; cyto-adherence and the inflammatory burst are the key events of pathogenesis in severe human malaria. In mice with *Plasmodium berghei* ANKA cerebral malaria, lovastatin reduces pro-inflammatory cytokines in the brain and prevents cognitive impairment [34].

Another hypothesis that could explain differences *in vitro* activity and *in vivo* efficacy is the role the cytochrome P450 enzyme. Most of the anti-malarial drugs are metabolised by the cytochrome P450 enzyme. Chloroquine, quinine, mefloquine, amodiaquine, lumefantrine, piperaquine, artemisinin derivatives and atorvastatin are metabolised by CYP3A4/A5 [35,36]. Artesunate and artemisinin are also metabolised by CYP2A6 and CYP2B6 [35]. Dihydroartemisinin is metabolised by UGT1A9 and UGT2B7 [35]. In addition, chloroquine and amodiaquine are metabolised by CYP2C8 [35]. Atorvastatin induces the activity of CYP2C8 and that of CYP3A4 but to a lesser way [37]. By increasing the P450 activity, atorvastatin could increase the catabolism of some anti-malarial drugs and reduce or sometimes increase (proguanil) the efficacy of the drug.

Even though *in vitro* data indicate that atorvastatin improved the activity of lumefantrine and piperaquine, the same may not necessarily be true *in vivo*. Piperaquine, a new drug with long terminal elimination half-life, is currently a very promising anti-malarial drug. These observations support calls for an *in vivo* evaluation of combination of atorvastatin with lumefantrine and piperaquine in a mouse model to treat uncomplicated malaria.

## Competing interests

All authors declare that they have no competing interests.

## Authors’ contributions

JD, HS, RA, and EB carried out the *in vitro* tests. BP conceived and coordinated the study. JD and BP analysed the data. JD and BP drafted the manuscript. All the authors read and approved the final manuscript.

## References

[B1] Le Bras J Musset L Clain J Antimalarial drug resistance Med Mal Infect 2006 36 401 405 16854546 10.1016/j.medmal.2006.05.005

[B2] Noedl H Se Y Schaecher K Smith BL Socheat D Fukuda MM Evidence of artemisinin-resistant malaria in western Cambodia N Engl J Med 2008 359 2619 2620 19064625 10.1056/NEJMc0805011

[B3] Dondorp AM Nosten F Yi P Das D Phyo AP Tarning J Lwin KM Ariey F Hanpithakpong W Lee SJ Ringwald P Silamut K Imwrong M Chotivanish K Lim P Herdman T An SS Yeung S Singhasivanon P Day NPJ Lindegardh N Socheat D White NJ Artemisinin resistance in *Plasmodium falciparum* malaria N Engl J Med 2009 361 455 467 19641202 10.1056/NEJMoa0808859 PMC3495232

[B4] Amaratunga C Sreng S Suon S Phelps ES Stepniewska K Lim P Zhou C Mao S Anderson JM Lindegardh N Jiang H Song J Su XZ White NJ Dondorp AM Anderson TCJ Fay MP Mu J Duong S Fairhurst RM Artemisinin-resistant *Plasmodium falciparum* in Pursat province, western Cambodia: a parasite clearance rate study Lancet Infect Dis 2012 12 851 858 22940027 10.1016/S1473-3099(12)70181-0 PMC3786328

[B5] White NJ Preventing antimalarial drug resistance through combinations Drug Resist Updat 2001 1 3 9 17092790 10.1016/s1368-7646(98)80208-2

[B6] Pradines B Torrentino-Madamet M Fontaine A Henry M Baret E Mosnier J Briolant S Fusai T Rogier C Atorvastatin is 10-fold more active in vitro than other statins against *Plasmodium falciparum* Antimicrob Agents Chemother 2007 51 2654 2655 17502414 10.1128/AAC.01330-06 PMC1913261

[B7] Parquet V Briolant S Torrentino-Madamet M Henry M Almeras L Amalvict R Baret E Fusai T Rogier C Pradines B Atorvastatin is a promising partner for antimalarial drugs in treatment of *Plasmodium falciparum* malaria Antimicrob Agents Chemother 2009 53 2248 2252 19307369 10.1128/AAC.01462-08 PMC2687225

[B8] Wurtz N Briolant S Gil M Parquet V Henry M Baret E Amalvict R Almeras L Rogier C Pradines B Synergy of mefloquine activity with atorvastatin, but not chloroquine and monodesethylamodiaquine, and association with the *pfmdr1* gene J Antimicrob Chemother 2010 65 1387 1394 20501488 10.1093/jac/dkq173

[B9] Parquet V Henry M Wurtz N Dormoi J Briolant S Gil M Baret E Amalvict R Rogier C Pradines B Atorvastatin as a potential anti-malarial drug: in vitro synergy in combinational therapy with quinine against *Plasmodium falciparum* Malar J 2010 9 139 20497586 10.1186/1475-2875-9-139 PMC2882376

[B10] Savini H Souraud JB Briolant S Baret E Amalvict R Rogier C Pradines B Atorvastatin as a potential antimalarial drug: in vitro synergy in combinational therapy with dihydroartemisinin Antimicrob Agents Chemother 2010 54 966 967 19949060 10.1128/AAC.01006-09 PMC2812141

[B11] Dormoi J Pascual A Briolant S Amalvict R Charras S Baret E Huyghues des Etages E Feraud M Pradines B Proveblue (Methylene Blue) as antimalarial agent: *In vitro* synergy with dihydroartemisinin and atorvastatin Antimicrob Agents Chemother 2012 56 3467 3469 22391526 10.1128/AAC.06073-11 PMC3370790

[B12] Borek-Dohalsky V Huclova J Barrett B Nemec B Ulc I Jelinek I Validated HPLC-MS-MS method for simultaneous determination of atorvastatin and 2-hydroxyatorvastatin in human plasma-pharmacokinetic study Anal Bioanal Chem 2006 386 275 285 16924379 10.1007/s00216-006-0655-3

[B13] Bienvenu AL Picot S Statins alone are ineffective in cerebral malaria but potentiate artesunate Antimicrob Agents Chemother 2008 52 4203 4204 18779350 10.1128/AAC.00513-08 PMC2573154

[B14] Helmers AJ Gowda DC Kain KC Liles WC Statins fail to improve outcome in experimental cerebral malaria and potentiate Toll-like receptor-mediated cytokine production by murine macrophages Am J Trop Med Hyg 2009 81 631 637 19815878 10.4269/ajtmh.2009.09-0204

[B15] Dormoi J Briolant S Desgrouas C Pradines B Impact of methylene blue and atorvastatin combination therapy on the apparition of cerebral malaria in a murine model Malar J 2013 12 127 23587099 10.1186/1475-2875-12-127 PMC3637457

[B16] Souraud JB Briolant S Dormoi J Mosnier J Savini H Baret E Amalvict R Soulard R Rogier C Pradines B Atorvastatin treatment is effective when used in combination with mefloquine in an experimental cerebral malaria murine model Malar J 2012 11 13 22233563 10.1186/1475-2875-11-13 PMC3278339

[B17] Dormoi J Briolant S Pascual A Desgrouas C Travaillé C Pradines B Improvement of the efficacy of dihydroartemisinin with atorvastatin in an experimental cerebral malaria murine model Malar J 2013 12 302 23988087 10.1186/1475-2875-12-302 PMC3765719

[B18] Lambros C Vanderberg JP Synchronization of *Plasmodium falciparum*erythrocytic stages in culture J Parasitol 1979 65 418 420 383936

[B19] Bogreau H Renaud F Bouchiba H Durand P Assi SB Henry MC Garnotel E Pradines B Fusai T Wade B Adehossi E Parola P Kamil MA Puijalon O Rogier C Genetic diversity and structure of African *Plasmodium falciparum* populations in urban and rural areas Am J Trop Med Hyg 2006 74 953 959 16760503

[B20] Henry M Diallo I Bordes J Ka S Pradines B Diatta B M'Baye PS Sane M Thiam M Gueye PM Wade B Touze JE Debonne JM Rogier C Fusai T Urban malaria in Dakar, Senegal: chemosusceptibility and genetic diversity of *Plasmodium falciparum* isolates Am J Trop Med Hyg 2006 75 146 151 16837722

[B21] Henry M Alibert S Baragatti M Mosnier J Baret E Amalvict R Legrand E Fusai T Barbe J Rogier C Pagès JM Pradines B Dihydroanthracene derivatives reverse in vitro quinoline resistance in *Plasmodium falciparum* malaria Med Chem 2008 4 426 437 18782039 10.2174/157340608785700234

[B22] Dokomajilar C Nsobya SL Greenhouse B Rosenthal PJ Dorsey G Selection of *Plasmodium falciparum pfmdr1* alleles following therapy with artemether-lumefantrine in an area of Uganda where malaria is highly endemic Antimicrob Agents Chemother 2006 50 1893 1895 16641472 10.1128/AAC.50.5.1893-1895.2006 PMC1472234

[B23] Lim P Akler AP Khim N Shah NK Incardona S Doung S Yi P Bouth DM Bouchier C Puijalon OM Meshnick SR Wongsrichanalai C Fandeur T Le Bras J Ringwald P Ariey F *Pfmdr1* copy number and artemisinin derivatives combination therapy failure in falciparum malaria in Cambodia Malar J 2009 8 11 19138391 10.1186/1475-2875-8-11 PMC2627910

[B24] Price RN Uhlemann AC Brockman A McReady R Ashley E Phaipun L Patel R Laing K Looareesuwan S White NJ Nosten F Krishna S Mefloquine resistance in *Plasmodium falciparum* and increased *pfmdr1* gene copy number Lancet 2004 364 438 447 15288742 10.1016/S0140-6736(04)16767-6 PMC4337987

[B25] Sidhu ABS Uhlmann AC Valderramos SG Valderramos JC Krishna S Fidock DA Decreasing *pfmdr1* copy number in *Plasmodium falciparum* malaria heightens susceptibility to mefloquine, lumefantrine, halofantrine, quinine, and artemisinin J Infect Dis 2006 194 528 535 16845638 10.1086/507115 PMC2978021

[B26] Holtzman CW Wiggins BS Spinler SA Role of P-glycoprotein in statin drug interactions Pharmacotherapy 2006 26 1601 1607 17064205 10.1592/phco.26.11.1601

[B27] Wang E Casciano CN Clement RP Johnson WW HMG-CoA reductase inhibitors (statins) characterized as direct inhibitors of P-glycoprotein Pharm Res 2001 18 800 806 11474784 10.1023/a:1011036428972

[B28] Wu X Whitfield LR Stewart BH Atorvastatin transport in the Caco-2 cell model: contributions of P-glycoprotein and the proton-monocarboxylic acid co-transporter Pharm Res 2000 17 209 215 10751037 10.1023/a:1007525616017

[B29] Pascual A Madamet M Bertaux L Amalvict R Benoit N Travers D Cren J Taudon N Rogier C Parzy D Pradines B *In vitro* piperaquine susceptibility is not associated with *Plasmodium falciparum*chloroquine resistance transporter gene Malar J 2013 12 431 24274185 10.1186/1475-2875-12-431 PMC4225597

[B30] Hao M Jia D Li Q He Y Yuan L Xu S Chen K Wu J Shen L Sun L Zhao H Yang Z Cui L *In vitro* sensitivities of *Plasmodium falciparum* isolates from the China-Myanmar border to piperaquine and association with polymorphisms in candidate genes Antimicrob Agents Chemother 2013 57 1723 1729 23357760 10.1128/AAC.02306-12 PMC3623367

[B31] Lim P Dek D Try V Eastman RT Chy S Sreng S Suon S Mao S Sopha C Sam B Ashley EA Miotto O Dondorp AM White NJ Su X-Z Chuor Char M Anderson JM Amaratunga C Menard D Fairhurst RM *Ex vivo* susceptibility of *Plasmodium falciparum* to antimalarial drugs in Western, Northern, and Eastern Cambodia, 2011–2012: Association with molecular markers Antimicrob Agents Chemother 2013 57 5277 5283 23939897 10.1128/AAC.00687-13 PMC3811250

[B32] Okombo J Abdi AI Kiara SM Mwai L Pole L Sutherland CJ Nzila A Ochola-Oyier LI Repeat polymorphisms in the low-complexity regions of *Plasmodium falciparum* ABC transporters and associations with *in vitro* antimalarial responses Antimicrob Agents Chemother 2013 57 6196 6204 24080667 10.1128/AAC.01465-13 PMC3837885

[B33] Taoufiq Z Pino P N'Dilimabaka N Arrouss I Assi S Soubrier F Rebollo A Mazier D Atorvastatin prevents *Plasmodium falciparum* cytoadherence and endothelial damage Malar J 2011 10 52 21356073 10.1186/1475-2875-10-52 PMC3056843

[B34] Reis PA Estato V da Silva TI d'Avila JC Siqueira LD Assis EF Bozza PT Bozza FA Tibiriça EV Zimmerman GA Castro-Faria-Neto HC Statins decrease neuroinflammation and prevent cognitive impairment after cerebral malaria PLoS Pathog 2012 8 e1003099 23300448 10.1371/journal.ppat.1003099 PMC3531520

[B35] Kerb R Fux R Morike K Kremsner PG Gil JP Gleiter CH Schwab M Pharmacogenetics of antimalarial drugs: effect on metabolism and transport Lancet 2009 9 760 774 19926036 10.1016/S1473-3099(09)70320-2

[B36] Lee TM Huang L Johnson MK Lizak P Kroetz D Aweeka F Parikh S In vitro metabolism of piperaquine is primarily mediated by CYP3A4 Xenobiotica 2012 42 1088 1095 22671777 10.3109/00498254.2012.693972 PMC5087332

[B37] Fiedt DM Klein K Hofmann U Riedmaier S Knobeloch D Thasler WE Weiss TS Schwab M Zanger UM Profiling induction of cytochrome P450 enzyme activity by statins using a new liquid chromatography-tandem mass spectrometry coktail assay in human hepatocytes Drug Metabolism Disposition 2010 38 1589 1597 20551241 10.1124/dmd.110.033886

